# Transformation of Transient Overvoltages by Inductive Voltage Transformers

**DOI:** 10.3390/s21124167

**Published:** 2021-06-17

**Authors:** Michał Kaczmarek, Dariusz Brodecki

**Affiliations:** Institute of Mechatronics and Information Systems, Lodz University of Technology, 90-924 Lodz, Poland; dariusz.brodecki@p.lodz.pl

**Keywords:** inductive voltage transformer, overvoltage, slow-front transient, ratio error, phase displacement, harmonics, distorted voltage

## Abstract

Overvoltage transients occur after any type of switching activity in a power network, such as breaker operation, fault occurrence/clearance and rapid load change. This distortion of voltage is transformed to the secondary circuit of a voltage transformer. The maximum values of such impulses may many times exceed the rated value of its secondary voltage. This can lead to malfunction of measuring or protection devices connected to the secondary circuit of a voltage transformer and even their damage. The paper presents the application of determined values of ratio error at harmonics of the inductive voltage of the transformer to predict the value of transformed slow-front transient overvoltage to their secondary circuits. This will help to prevent malfunction of measuring or protection devices connected to the secondary side of the voltage transformer and increase their safety of operation. The inductive voltage transformer equivalent circuit for transformation of higher frequency components of distorted voltage must be extended with internal capacitances of windings. This is caused by the fact that the resonance phenomenon of the slow-front transient overvoltage results from leakage inductance and capacitance of primary winding, not from the magnetic core. Therefore, this behaviour is independent from the value of the applied voltage.

## 1. Introduction

**Figure 1:**
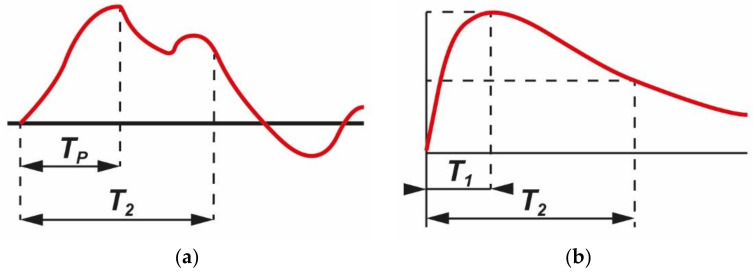
IEC 60071-1: (**a**) slow-front transient overvoltage, and (**b**) test voltage [1].

In accordance with the standard IEC 60071-1, transient overvoltages may be divided into three types: slow-front, fast-front and very fast-front [1,2]. It applies to three-phase AC systems having a highest voltage for equipment above 1 kV. In the power networks slow front transient overvoltage could be produced by load rejection or phase to ground faults. One of the principal causes of fast front overvoltages in power systems is a lightning strike to transmission lines. Very fast transient overvoltage could be generated in a gas insulated substation (GIS) during the opening and closing of circuit breakers or disconnectors [3,4]. In the standard IEC 60071-1 the procedure for the selection of the rated withstand voltages of the equipment and the installations of these systems are specified [1]. The values of overvoltage that stress the insulation are required to be determined in amplitude, shape and duration. For each type, different shapes of test voltages are defined. The shapes of slow-front transient overvoltage (a) and defined test voltage (b) are presented in Figure 1.

In Figure 1 the following notations are used:

T_p_—time to peak value: (a) 20 µs < T_p_ ≤ 5000 µs,

T_1_—front time: (b) 250 µs,

T_2_—time to the half value of a decreasing voltage: (a) T_2_ ≤ 20 ms, b) T_2_ = 2500 µs.

In the case of the fast-front overvoltage, its front time T_1_ is defined to be between 0.1 and 20 µs; in the test voltage it is required to be equal to 1.2 µs, while its time to half value of a decreasing voltage is below or equal to 300 µs, and in the test voltage it is required to be equal to 50 µs [1]. The assumed maximum values of the representative overvoltages of the slow-front or fast-front types result from combined voltages consisting of a standard switching impulse and of a power-frequency voltage, each with peak value equal to the two relevant assumed maximum peak values.

In accordance with the standard IEC 61869-1 to test overvoltage transformed by the voltage transformer, a voltage impulse is applied between one of the primary terminals and earth [5]. This special test is defined for units having highest voltage for equipment equal or higher to 72.5 kV. Two types of the voltage wave shape may be applied. Wave shape A is used for equipment intended to be mounted in an air-insulated switchgear. Wave shape B is used for equipment intended to be mounted on a gas-insulated switchgear. The peak value of the test voltage *U_1_* shall have a value between 50 kV and the reference voltage *U_pref_* resulting from the equation:

(1) $$U_{pref}=1.6\cdot \frac{\surd 2\cdot U_{m}}{\surd 3}$$

where *U_m_* is the highest voltage for equipment: the greatest value of phase-to-phase voltage for which the equipment is designed in respect of its insulation as well as other characteristics which relate to this voltage in the relevant equipment standards under normal service conditions [5].

Wave shape A is similar to that presented in Figure 1b, and is characterized by front time 0.5 μs ± 0.1 µs and time to half-value equal or greater than 50 μs. Due to these parameters this impulse is not equivalent to the exposure to slow-front overvoltage transients. From Equation (1) it follows that the peak value of the reference voltage should be equal to about 32 kV peak for the voltage transformer having highest voltage for equipment equal to 24 kV. The overvoltage transmitted to the secondary winding for the reference overvoltage applied to the primary winding is calculated as follows [5]:

(2) $$U_{TOV}=U_{pref}\cdot \frac{U_{2}}{U_{1}}$$

The peak value of the overvoltage transmitted the secondary terminals shall not exceed 1.6 kV under the test and measuring conditions.

The inductive instrument transformers are subject to many nonlinear effects causing problems in the accurate measurement of PQ issues [4,6,7,8,9,10,11,12]. The rms values of the slow-front transient overvoltage in the secondary circuit of the inductive voltage transformer results from the internal resonance caused by the simultaneous presence of inductances and capacitances in the inductive voltage transformer (VT) [6,7,12,13,14,15,16]. In this paper the determined characteristic of voltage ratio error at harmonics with frequency for a given medium voltage inductive instrument transformer is used to predict the value of the slow-front overvoltage in its secondary circuit. The nonlinearity of the magnetization characteristics of the magnetic core of the inductive VTs causes the need for accurate evaluation of the values of ratio error and phase displacement at harmonics of the rated primary voltage; this is required during the test [17,18,19,20,21,22,23,24]. There are many methods and measuring systems that enable evaluation of the values of ratio error at harmonics of inductive VT. The most recent solutions from a given group of authors are referenced as [17,18,25]. However, the resonance frequencies and the measured values of ratio error and phase displacement do not change with the value of the applied voltage. This is due to the fact that the transformation accuracy of the slow-front transient overvoltages results from the leakage inductances and the capacitances of windings [14,15,16,19,20]. It should be noted that at the rated voltage transient overvoltages are more likely to cause ferroresonance [13]. The objects of the research are dual pole VTs, two with rated primary voltage equal to 20 kV and one with rated primary voltage equal to 15 kV. Their properties are studied under switching overvoltages caused by failure in operation of the Pulse-Width Modulation (PWM) based programmable AC source when used to supply a step-up transformer [26].

## 2. Tested Objects and Their Equivalent Circuit

Two VTs with rated primary voltage equal to 20 kV are characterized by the maximum insulation level (24/50/125) kV and the 15 kV type is declared by manufacturer as (17.5/38/95) kV. Their rated secondary voltage is equal to 100 V. Their accuracy class is 0.5 and the rated apparent power of the secondary winding is 25 VA.

**Figure 2:**
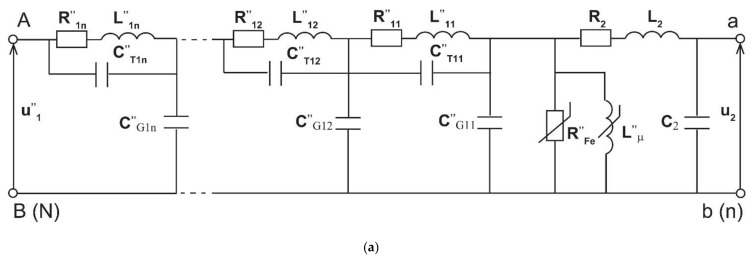

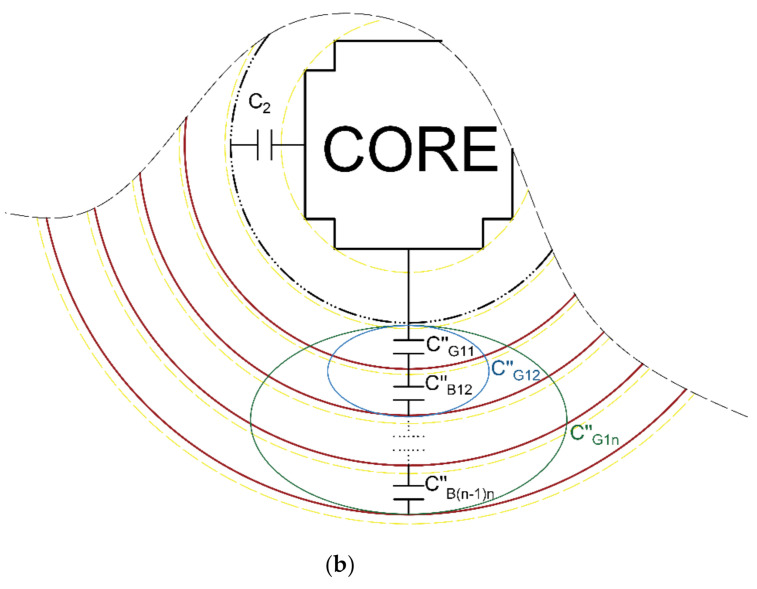
(**a**) Equivalent circuit of the inductive VT for transformation of the conductive disturbances of frequencies up to 20 kHz, and (**b**) cross-section of the primary winding with capacitances.

To analyse the transformation of conducted disturbances by inductive VTs an extended equivalent circuit is used (Figure 2) [14]. It also includes the partial equivalent capacitances of the windings and between the windings of the inductive VT.

In Figure 2 the following notations are used—symbols with two dashes (‘’) indicate parameters converted to a secondary circuit: C”_T11_/C”_T12/_C”_T1n_—partial equivalent capacitance between the primary and secondary windings, C”_G11_/C”_G12/_C”_G1n_—partial equivalent capacitance of the primary winding to ground, C”_B12_/C”_B(n−1)n_—parasitic capacitance between neighbour layers of the primary winding, R”_11_/R”_12/_R”_1n—_partial resistance of the primary winding, L”_11/_L”_12/_L”_1n_—partial leakage inductance of the primary winding, C_2_—equivalent capacitance of the secondary winding, R_2_—resistance of the secondary winding, L_2_—leakage inductance of the secondary winding, R”_Fe_—equivalent resistance of the iron losses in the magnetic core, L”_μ_—mutual inductance between primary and secondary windings, u”_1_—instantaneous value of the primary voltage, u_2_— instantaneous value of the secondary voltage.

To correctly reproduce the multi-resonance circuit of the test object in the equivalent circuit, primary winding capacitance and leakage inductance are divided into several parts, as presented in paper [14]. Between each two layers of the primary winding (Figure 2b) the parasitic capacitance is present. The partial equivalent capacitance of the primary winding to ground (ground stray capacitance) forms the conductive path to ground of each layer of the primary winding. The parasitic capacitance is also present between turns of the windings. For each layer of the primary winding it forms the conductive path through the partial equivalent capacitance between the primary and secondary windings. The serial connection is equal to the equivalent capacitance between the primary and secondary windings. The thickness of the insulation layers between the magnetic core and the secondary winding, as well as between the windings, determines the internal coupling capacitance of the VT. They are selected in order to ensure immunity to voltage stress resulting from the maximum required value of the voltage between the insulated components and mainly from the rated rms value of the primary voltage. This capacitance is responsible for the value of the transfer coefficient of high frequency conducted disturbances. The increase of their frequency above about 20 kHz causes the parasitic capacitances to become the most important path of the coupling between primary and secondary windings. Moreover, the parasitic capacitances should be considered as distributed between all terminals of the inductive VT.

## 3. The Measuring System Used for Evaluation of the Transformation Accuracy of Slow-Front Transient Overvoltages

**Figure 3:**
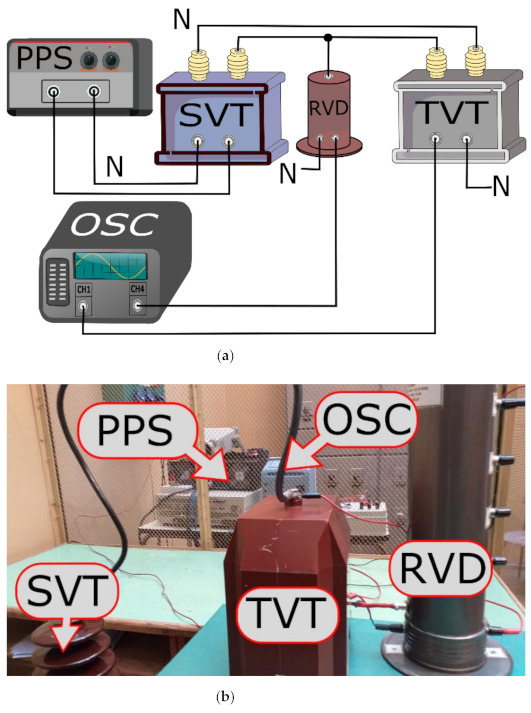
The measuring system used for evaluation of the transformation accuracy of slow-front transient overvoltages: (**a**) block diagram, and (**b**) photo of the test setup.

The evaluation of the transformation accuracy of slow-front transient overvoltages from primary to secondary circuits of tested VTs was made in the measurement system presented in Figure 3.

In Figure 3 the following notations are used: PPS—programmable AC power source, SVT—step-up transformer, TVT—tested inductive VT, RVD—reference wideband voltage divider, OSC—oscilloscope.

The measuring system was supplied from the PWM based programmable AC power source [26] The step-up transformer was used to increase its output voltage to 2 kV or 1.5 kV RMS. These values are equal to 10% of the rated RMS value of the primary voltage of the tested inductive VT. Such reduction is required to avoid damaging the measuring system by a high peak value of the transient overvoltage. Laboratory tests consisted of simultaneous observation, using an oscilloscope, of voltage waveforms on the primary and secondary side of the tested VT. Primary voltage was measured with the medium voltage (MV) divider with input voltage 2 kV used as the high voltage probe [20].

## 4. The Measuring System Used to Determine the Transformation Accuracy of Harmonics of Distorted Primary Voltage

**Figure 4:**
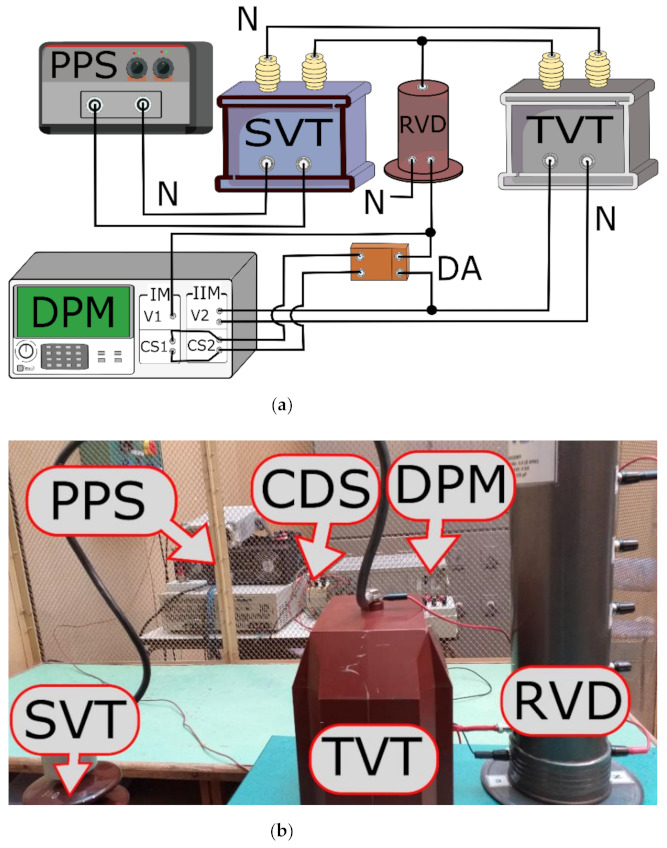
The measuring system used to determine the transformation accuracy of harmonics of distorted primary voltage: (**a**) block diagram, and (**b**) photo of the test setup.

The values of voltage ratio error and phase displacement of tested VTs for transformation of distorted voltage’s harmonics were determined in the measuring system presented in Figure 4 [19,20,23].

In Figure 4 the following new notations are used: CDS—converter of differential voltage to single ended voltage, DPM—digital power meter.

The wideband voltage divider with multiple input voltages 15 and 20 kV was used as the source of the reference voltage. Output voltage of the divider and output voltage of the wideband high impedance converter of differential voltage to single ended voltage are simultaneously measured by two voltage channels of the digital power meter. Implemented in this device is a Fast Fourier Transform algorithm that enables determination of up to the 100th harmonic. The differential voltage is measured between the high potential terminal of the tested VT’s secondary winding and high potential terminal of the output of the reference voltage divider. This enabled calculation of the voltage composite error and the value of the secondary voltage of the tested VT. Then the values of the voltage ratio error and the phase displacement of harmonics transformation may be determined. To supply the measuring circuit the step-up transformer and the programmable AC power source were used. This enabled generation of the distorted primary voltage with the fundamental component of frequency equal to 50 Hz That rms value may achieve 20 kV with higher harmonics (up to 100-th); that rms value may be set up to 10% of the main harmonic [26].

The voltage ratio error of transformation of the distorted voltage’s k-order harmonic expressed as a percentage of the primary voltage is given by the formula [25]:

(3) $$∆U_{TVThk}=\frac{U_{TVThk}-U_{RVDhk}}{U_{RVDhk}}\cdot 100\%$$

where *U_TVTkh_*—the rms value of the k-order harmonic of the distorted secondary voltage of the tested VT, and *U_RVDkh_*—the rms value of the k-order harmonic of voltage of the reference voltage divider.

The secondary voltage of the tested VT is calculated from the following equation [25]:

(4) $$\;U_{TVThk}=\sqrt{U_{RVDhk}^{2}+U_{CDShk}^{2}-2\cdot U_{RVDhk}\cdot U_{CDShk}\cos\left(180-\varphi_{RVD\_CDShk}\right)}$$

where *U_TVThk_*—the RMS values of the k-order harmonic in the secondary voltage of tested VT, *U_RVDhk_*—the RMS values of the k-order harmonic in the output voltage of the reference voltage divider, *U_CDShk_*—the RMS values of the k-order harmonic in the output voltage of the converter of differential voltage to single ended voltage, *φ_RVD_CDS_*—the phase shift of the k-order harmonic in the output voltage of the converter in relation to the same frequency harmonic in the reference voltage from the divider.

The composite error of transformation of the hk-order harmonic of the distorted voltage by tested VT expressed in percentages of the primary voltage is calculated from the following equation [25]:

(5) $${∆\varepsilon }_{VThk}=\frac{U_{CDShk}}{U_{VThk}}\cdot 100\%$$

If the value of the composite error is determined, the value of the phase displacement at harmonics may be calculated from the following formula [25]:

(6) $${\delta U}_{TVThk}=\arcsin\left(\frac{\sqrt{{∆\varepsilon }_{VThk}^{2}-{∆U}_{TVThk}^{2}}}{100\%}\right)$$

**Figure 5:**
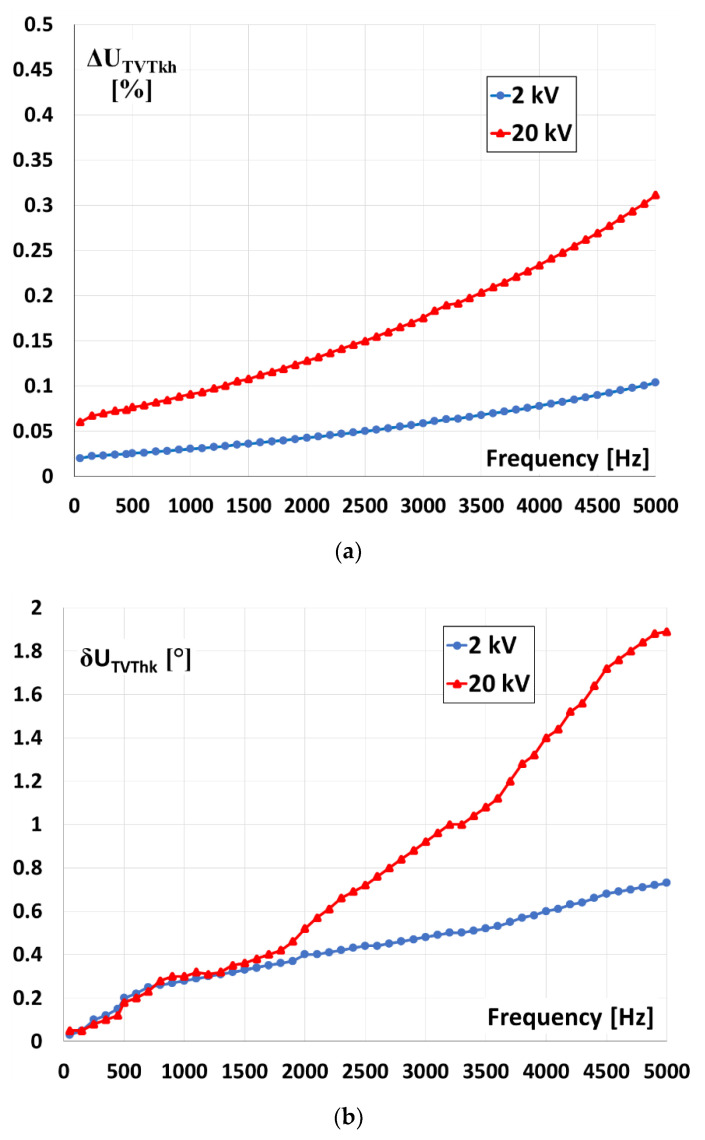
Voltage ratio error (**a**) and phase displacement (**b**) of used 2 kV and 20 kV (15 V) voltage dividers determined for sinusoidal voltage 200 V.

The wideband voltage dividers used for evaluation of the transformation accuracy of VTs at harmonics of distorted primary voltage in measuring systems presented in Figure 4 and Figure 5 were tested as described in the paper [27] and in accordance with the requirements of the standard [28]. The division accuracy and phase linearity of voltage dividers are characterized by voltage ratio error and phase displacement measured for sinusoidal input voltage equal to 200 V RMS. In Figure 5 the results are presented for voltage dividers with input voltage equal to 2 kV and 20 kV (15 kV).

The results obtained for the voltage divider with its input voltage equal to 15 kV are very similar to those presented for its 20 kV input. Taking into consideration the measurement uncertainty, the values of voltage ratio error and phase displacement of the voltage divider with the input voltage equal to 2 kV are ±0.05%/±0.1 for 50 Hz and increase to ±0.5%/±1.0 for 5 kHz. In the case of the voltage divider with input voltages equal to 20 kV and 15 kV it is ±0.1%/±0.1 for 50 Hz and increase to ±1.0%/±2.5 for 5 kHz.

The measurement uncertainty of the differential measuring system used for evaluation of the values of voltage ratio error and phase displacement of tested VTs for transformation of distorted voltage’s harmonics (Figure 4) is analysed in detail in the paper [25].

## 5. The Results of Evaluation of the Transformation Accuracy of the Slow-Front Transient Overvoltages

**Figure 6:**
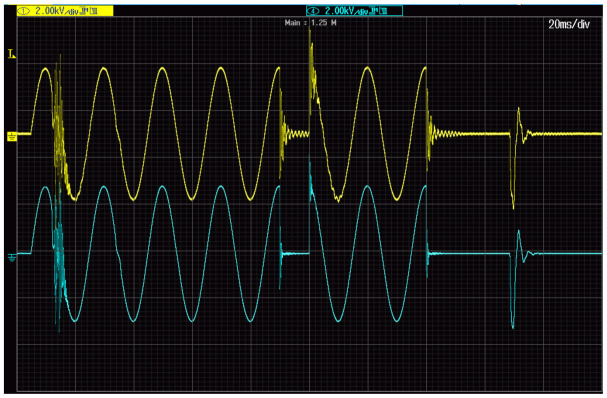
Voltage waveforms of voltages on primary (blue line) and secondary (yellow line) windings of the 2 kV step-up transformer.

Figure 6 shows the voltage waveform used for evaluation of the transformation accuracy of the slow-front transient overvoltages by inductive VTs. Their properties are studied under switching overvoltages cause by failure in operation of the PWM based programmable AC source when used to generate a voltage dip. This event is caused by inductive load resulting from the step-up transformer.

The RMS value of the secondary voltage of the step-up transformer is equal to 2 kV, while its primary voltage is equal to 100 V RMS. In the next cases only the part of the waveforms with slow-front transient overvoltages are analysed.

**Figure 7:**
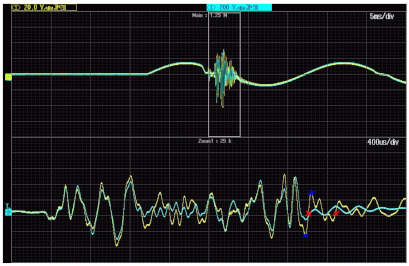
Voltage waveforms of voltages on primary (blue line) and secondary (yellow line) windings of the 20 kV inductive VT (model 1).

Figure 7 shows the voltage waveforms on primary and secondary windings of the first tested 20 kV inductive VT. The RMS value of the primary voltage is equal to 2 kV.

The maximum value of the overvoltage on the primary winding of the 20 kV inductive VT (model 1) achieved about 10 kV for 2 kV RMS value of the primary voltage. The frequency response of the VT does not cause significant increase of this value. The change of the frequency of oscillations to about 3.1 kHz (320 μs period between red crosses) causes the overvoltage value in the primary circuit to be equal to about 1.4 kV and the overvoltage in the secondary circuit of VT, equal to about 4.8 kV (half of the peak to peak value between blue crosses). Therefore, as results from Equation (3) show, the ratio error reaches +240%.

**Figure 8:**
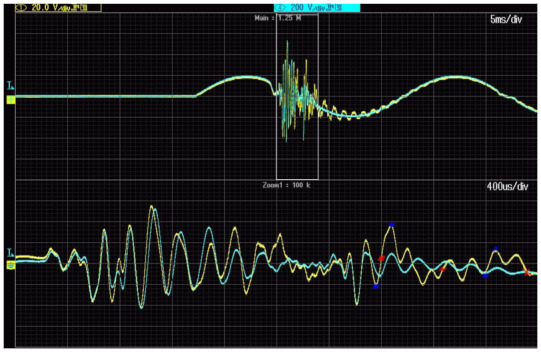
Voltage waveforms of voltages on primary (blue line) and secondary (yellow line) windings of the 20 kV voltage transformer (model 2).

Figure 8 shows the voltage waveforms on the primary and secondary windings of the second tested 20 kV inductive VT. The RMS value of the primary voltage is equal to 2 kV.

The maximum value of the overvoltage on the primary winding of the 20 kV inductive VT (model 2) achieved again about 10 kV for 2 kV RMS value of the primary voltage. The frequency response of the VT does not cause a significant increase in this case. If the frequency of oscillation reaches about 1.5 kHz (680 μs period between the second and third red cross from the left), the value of overvoltage in the primary circuit is equal about 1.2 kV, while the overvoltage in the secondary circuit of VT is equal to about 4.0 kV (half of the peak to peak value between blue crosses). Therefore, as results from Equation (3) show, the ratio error reaches +230%. If the frequency of oscillation reaches about 2.3 kHz (440 μs period between the first and second red cross) the value of overvoltage in the primary circuit is equal to about 2.0 kV, while the overvoltage in the secondary circuit of VT is equal to about 5.2 kV (half of the peak to peak value between blue crosses). Therefore, as results from Equation (3) show, the ratio error reaches +160%.

**Figure 9:**
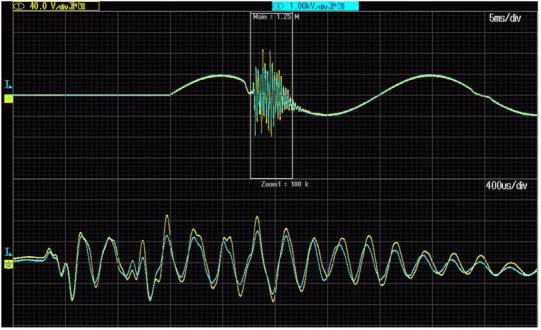
Voltage waveforms of voltages on primary (blue line) and secondary (yellow line) windings of the 15 kV voltage transformer.

Figure 9 shows the voltage waveforms on the primary and secondary windings of the 15 kV inductive VT. The RMS value of the primary voltage is equal to 1.5 kV.

The maximum value of the overvoltage on the primary winding of the 15 kV inductive VT achieved about 4 kV for 1.5 kV RMS value of the primary voltage. The frequency response of the VT does not cause significant increase of any oscillations.

## 6. The Results of Evaluation of the Transformation Accuracy of Harmonics of Distorted Primary Voltage

**Figure 10:**
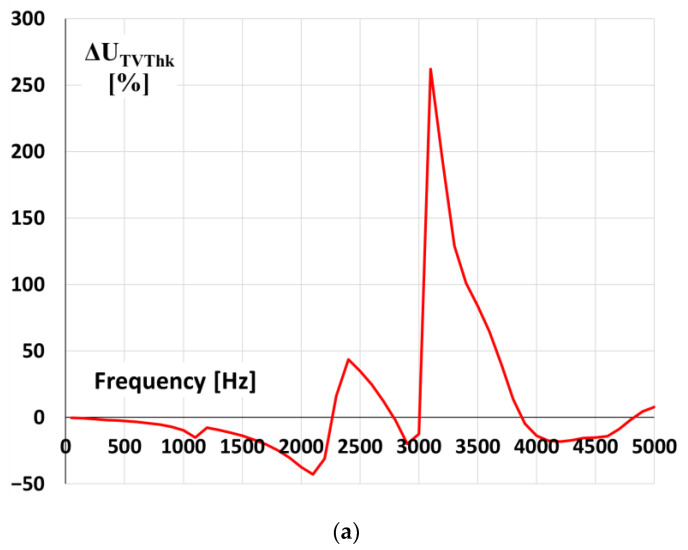

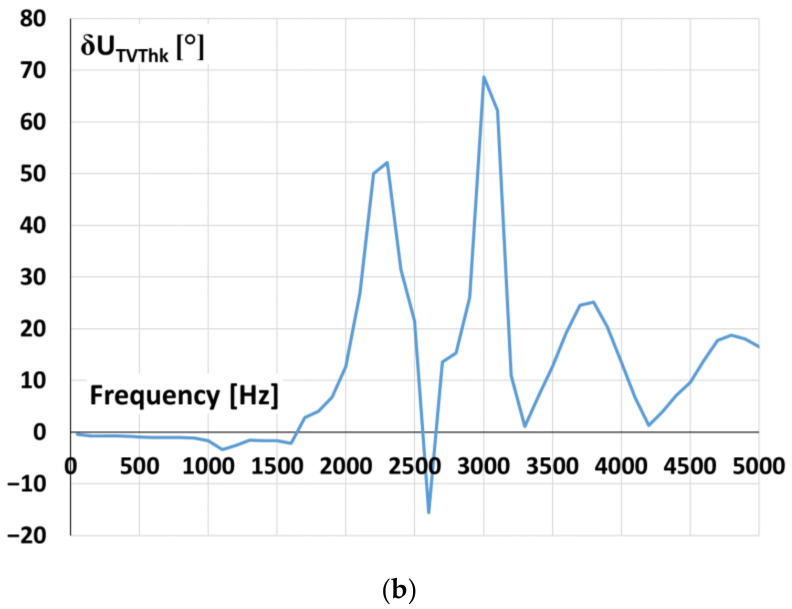
The values of (**a**) voltage error and (**b**) phase displacement at harmonics of the 20 kV voltage transformer (model 1).

The values of the voltage ratio error and phase displacement of the first tested inductive VT with rated primary voltage equal to 20 kV for transformation of the distorted voltage’s harmonics are presented in Figure 10.

The values of voltage error and phase displacement at harmonics are determined for distorted primary voltage equal to 20 kV RMS with a 10% level of tested higher harmonic of selected frequency from 100 Hz (2nd harmonic for the 50 Hz main frequency) up to 5 kHz (100th harmonic). It results from Figure 10a; that high overvoltage is expected at frequencies of higher harmonic between 3.1 and 3.4 kHz. Therefore, the frequency of oscillation equal to about 3.1 kHz (Figure 7) caused an overvoltage value in the primary circuit equal to about 1.2 kV the overvoltage in the secondary circuit of VT, equal to about 4.8 kV and the value of the ratio error reached +240%. The difference between the determined value of ratio error in Figure 10a results mainly from the measurement uncertainty of determination of oscillation frequency from the voltage waveform and its RMS value by the oscilloscope.

**Figure 11:**
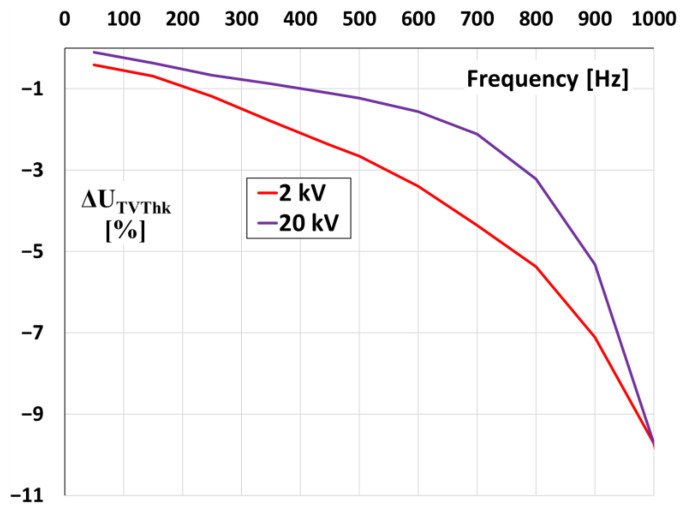
The values of voltage error at harmonics of the 20 kV voltage transformer (model 1) for different levels of primary voltage with a 10% higher harmonic.

In Figure 11 the values of voltage error at harmonics of the 20 kV voltage transformer (model 1) for two RMS levels of primary voltage 20 and 2 kV are presented.

The nonlinearity of the magnetization characteristics of the magnetic core of the inductive VT causes that for accurate evaluation of the values of ratio error and phase displacement at harmonics the rated primary voltage is required during the test. However, the resonance frequencies and the ratio error are not changed. This is caused by the fact that the resonance phenomenon of the slow-front transient overvoltage results from leakage inductance and capacitance of primary winding not from the magnetic core. Therefore, this behaviour is independent from the value of the applied voltage.

**Figure 12:**
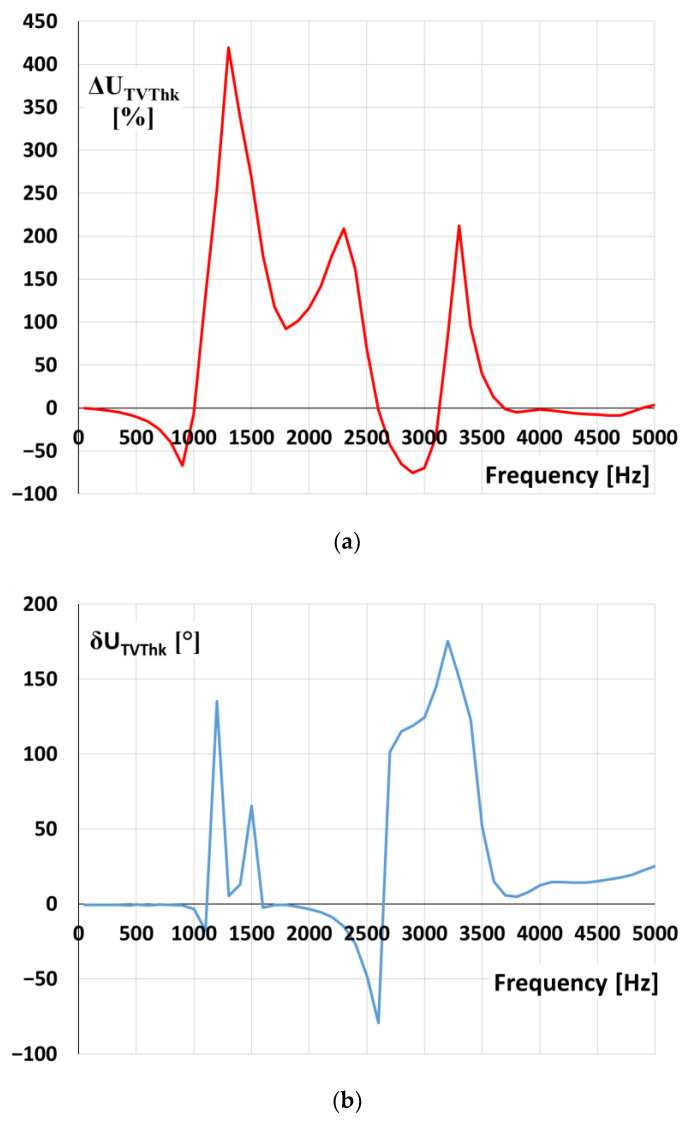
The values of (**a**) voltage error and (**b**) phase displacement at harmonics of the 20 kV voltage transformer (model 2).

The values of voltage ratio error and phase displacement of second tested inductive VT with rated primary voltage equal to 20 kV for transformation of distorted voltage’s harmonics are presented in Figure 12.

Results from Figure 12a show that high overvoltages are expected at frequencies of higher harmonic from 1.1 to 1.6 kHz and from 2.0 to 2.4 kHz, as well as from 3.1 to 3.4 kHz. Therefore, for the frequency of oscillation equal to about 1.5 kHz, the value of overvoltage in the primary circuit is equal to about 1.2 kV, while the over-voltage in secondary circuit of VT is equal about 4.0 kV. Then, the ratio error reaches +230% (Figure 8), and the expected value was 250% (Figure 12a). If the frequency of oscillation is about 2.3 kHz, the value of overvoltage in the primary circuit is equal to about 2.0 kV, while the overvoltage in the secondary circuit of VT is equal to about 5.2 kV. Therefore, the ratio error reaches +160%, and the expected value was 180% (Figure 12a). The difference between the determined value of the ratio error in Figure 8 and Figure 12a does not exceed 20% for both 1.5 and 2.3 kHz. Determined values of phase displacement at harmonics of the second tested 20 kV voltage transformer presented in Figure 12b confirms the resonance behaviour at expected frequencies.

**Figure 13:**
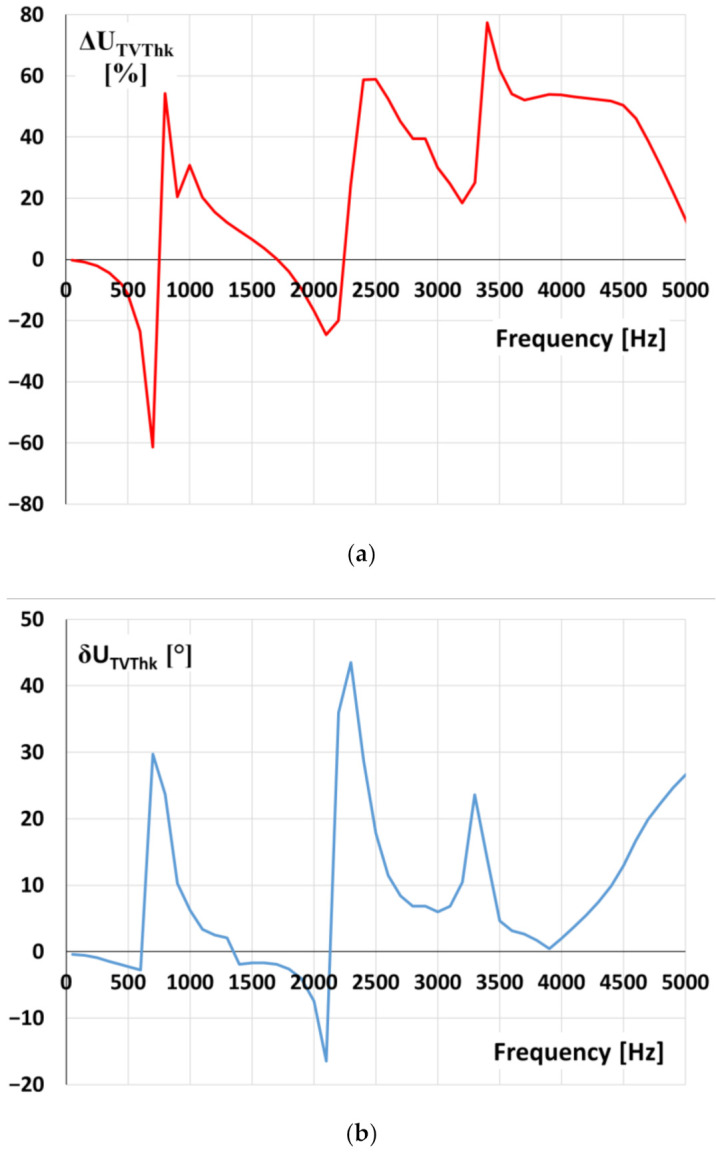
The values of the (**a**) voltage error and (**b**) phase displacement at harmonics of the 15 kV voltage transformer.

The values of the voltage ratio error and phase displacement of the tested inductive VT with rated primary voltage equal to 15 kV for transformation of the distorted voltage’s harmonics are presented in Figure 13.

The frequency response of the inductive VT with rated primary voltage equal to 15 kV shows high nonlinearity and also poor transformation accuracy of higher harmonics, but this unit is unlikely to increase the slow-front transient overvoltages. This expected behaviour is confirmed by results presented in Figure 9.

## 7. Conclusions

The good compliance of the results of evaluation of the transformation accuracy of harmonics of distorted primary voltage and the measurement results allows the conclusion that the peak value of the slow-front transient overvoltage in the secondary circuit of the MV inductive voltage transformer may be predicted from the determined values of the ratio error at harmonics. Therefore, application of the proposed solution may prevent a malfunction of measuring or protection devices connected to the secondary side of the voltage transformer and increase their safety of operation. The resonance phenomenon of the slow-front transient overvoltage results from leakage inductance and capacitance of primary winding. Therefore, the resonance behaviour of the inductive VT is independent from the value of the applied voltage. Moreover, inductive VTs with same rated primary voltage but from different manufacturers may be characterized by significantly different frequency response. It may be expected that with the decrease of the rated primary voltage of inductive VT the increase of slow-front transient overvoltage is less likely to occur.

## Data Availability

Not applicable.
